# Biomechanical evaluation of a novel minimally invasive pedicle bone cement screw applied to the treatment of Kümmel’s disease in porcine vertebrae

**DOI:** 10.3389/fbioe.2023.1218478

**Published:** 2023-07-05

**Authors:** Xiang Ma, Qing Feng, Xingze Zhang, Xiaolei Sun, Longwei Lin, Lin Guo, Lijun An, Shenglin Cao, Jun Miao

**Affiliations:** ^1^ Tianjin Hospital, Tianjin University, Tianjin, China; ^2^ Tianjin Medical University, Tianjin, China; ^3^ Chengde Medical College, Hebei, China

**Keywords:** Kümmel’s disease, osteoporosis, novel bone cement screw, biomechanics, porcine

## Abstract

**Background and objective:** Treatment of Kümmel’s Disease (KD) with pure percutaneous kyphoplasty carries a greater likelihood of bone cement displacement due to hardened bone and defect of the peripheral cortex. In this study, we designed a novel minimally invasive pedicle bone cement screw and evaluate the effectiveness and safety of this modified surgical instruments in porcine vertebrae.

**Methods:** 18 mature porcine spine specimens were obtained and soaked in 10% formaldehyde solution for 24 h. 0.5000 mmol/L EDTA-Na_2_ solution was used to develop *in vitro* osteoporosis models of porcine vertebrae. They were all made with the bone deficiency at the anterior edge of L1. These specimens were randomly divided into 3 groups for different ways of treatment: Group A: pure percutaneous kyphoplasty (PKP) group; Group B: unilateral novel minimally invasive pedicle bone cement screw fixation combined with PKP group; Group C: bilateral novel minimally invasive pedicle bone cement screw fixation combined with PKP group. The MTS multi-degree of freedom simulation test system was used for biomechanical tests, including axial loading of 500 N pressure, range of motion (ROM) in flexion, extension, left/right lateral bending, and left/right axial rotation at 5 Nm, and the displacement of bone cement mass at maximum angles of 5° and 10°.

**Result:** The three groups were well filled with bone cement, no leakage or displacement of bone cement was observed, and the height of the vertebrae was higher than pre-operation (*p* < 0.05). In the left/right axial rotation, the specimens were still significantly different (*p* < 0.05) from the intact specimens in terms of ROM after PKP. In other directions, ROM of all group had no significant difference (*p* < 0.05) and was close to the intact vertebrae. Compared with PKP group, the relative displacement of bone cement in groups B and C was smaller (*p* < 0.05).

**Conclusion:** In the *in vitro* animal vertebral models, the treatment of KD with the placement of novel pedicle minimally invasive bone cement screw combined with PKP can effectively restore the vertebral height, improve the stability of the affected vertebra and prevent the displacement of bone cement. Biomechanically, there is no significant difference between bilateral and unilateral fixation.

## 1 Introduction

Kümmel’s disease (KD), a rare type of osteoporotic vertebral compression fracture (OVCF), is characterized by an intravertebral vacuum cleft (IVC)in radiological imaging (Qi HR. et al., 2022; Gou et al., 2023). Patients with OVCF can experience relief of symptoms with non-surgical treatment, but about 1/3 of patients may have persistent low back pain and kyphosis, which may develop into KD (Duan et al., 2019). KD presents with back pain without obvious causes or after minor trauma based on osteoporosis, followed by a long or short asymptomatic period. Eventually, prolonged spine pain reappears in the same area and cannot be relieved, leading to the development of kyphosis (Qi J. et al., 2022; Li et al., 2023).

KD generally does not heal spontaneously, and alternative treatments such as bed rest and stent fixation have shown limited success for the treatment. They are often not sufficient to address the underlying bone deficiency and may lead to persistent symptoms and potential complications (Wang et al., 2020; Zhang et al., 2022). Moreover, patients are usually the elderly, and long-term bed rest can lead to various complications. Therefore, KD patients usually need surgical treatment such as percutaneous vertebroplasty (PVP) and percutaneous kyphoplasty (PKP) (Niu et al., 2017; Zhang et al., 2021). Internal fixation should also be considered when the patient’s kyphosis is significant and compresses the spinal cord or neural structures (Gan et al., 2021; Huang et al., 2021; Han et al., 2022). Compared with common OVCF, KD tends to have a longer course of disease and have a greater likelihood of bone cement displacement after surgery due to the presence of hardened bone and defect of the peripheral cortex on either side of the IVC (Chen et al., 2015; Li et al., 2017). The hardened bone makes it difficult for bone cement to penetrate the cancellous bone and form a uniform and cohesive mass within the vertebral body (Li et al., 2017; Liu JB. et al., 2022). Displacement of bone cement can lead to nerve and spinal cord damage (Yang et al., 2008).

**FIGURE 1:**
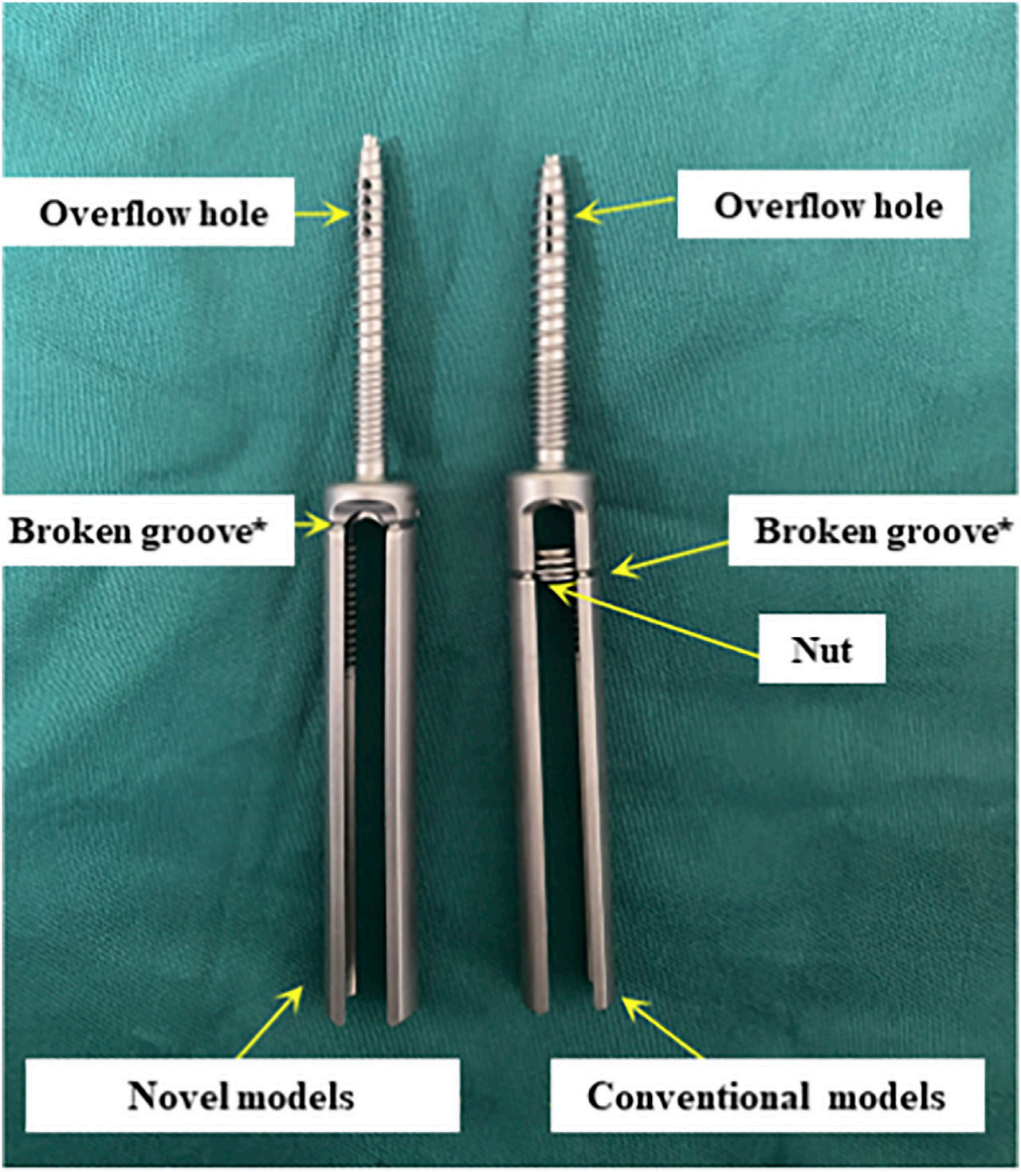
Novel and conventional cannulated pedicle screw. *, representing our improvements.

Currently, some studies have proposed cement augmentation combined with short segmental fixation to strengthen bone cement anchoring, but these methods are difficult to achieve minimally invasive treatment (Wang et al., 2021; Liu Y. et al., 2022; Zhang et al., 2022). The short segmental fixation could be not required for patients with KD without neurological compression symptoms. To address these clinical issues, we considered strengthening the interaction force between bone cement and bone tissue directly through pedicle screws in the treatment of KD with defect of the peripheral cortex. And the problem of wearing the muscles and other soft tissue due to the excess caudal design of the conventional pedicle bone cement screws should be further avoided (Figure 1). In this study, 18 *in vitro* specimens of KD were prepared to evaluate and analysis the effectiveness and safety of fixing bone cement mass using this modified surgical instrument.

## 2 Materials and methods

### 2.1 Material selection and preparation

**FIGURE 2:**
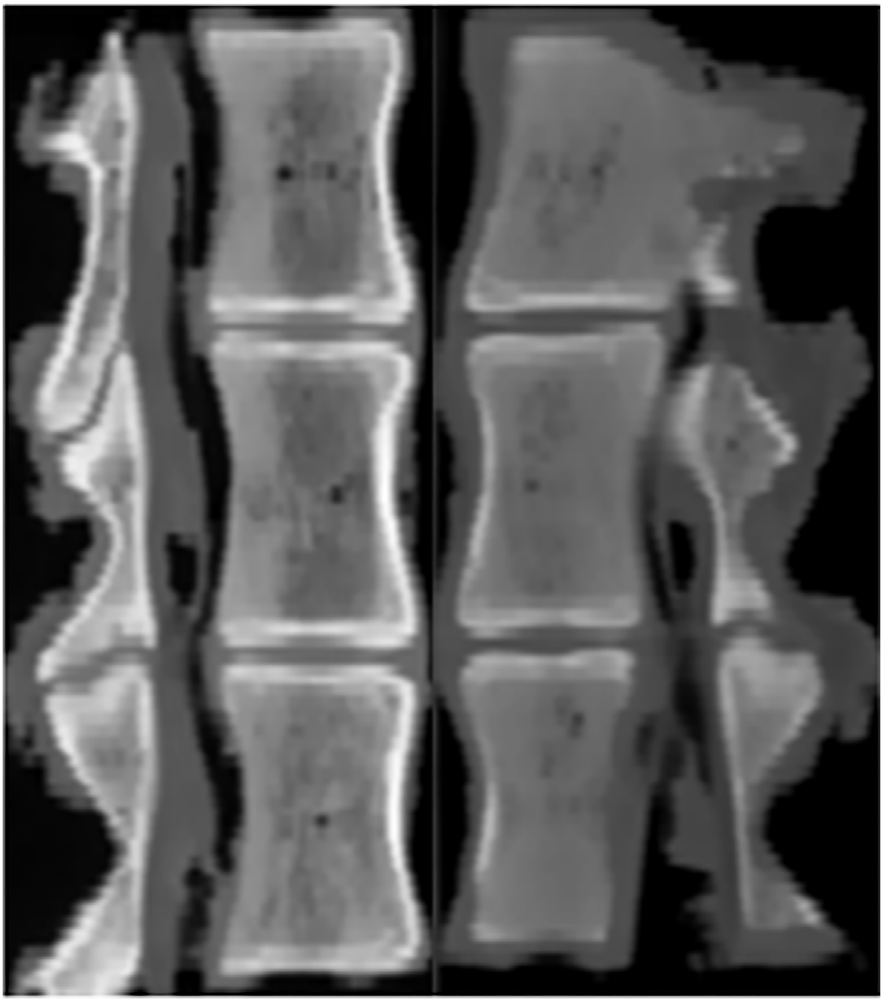
Sagittal reconstruction CT images before (left) and after (right) decalcification.

**FIGURE 3:**
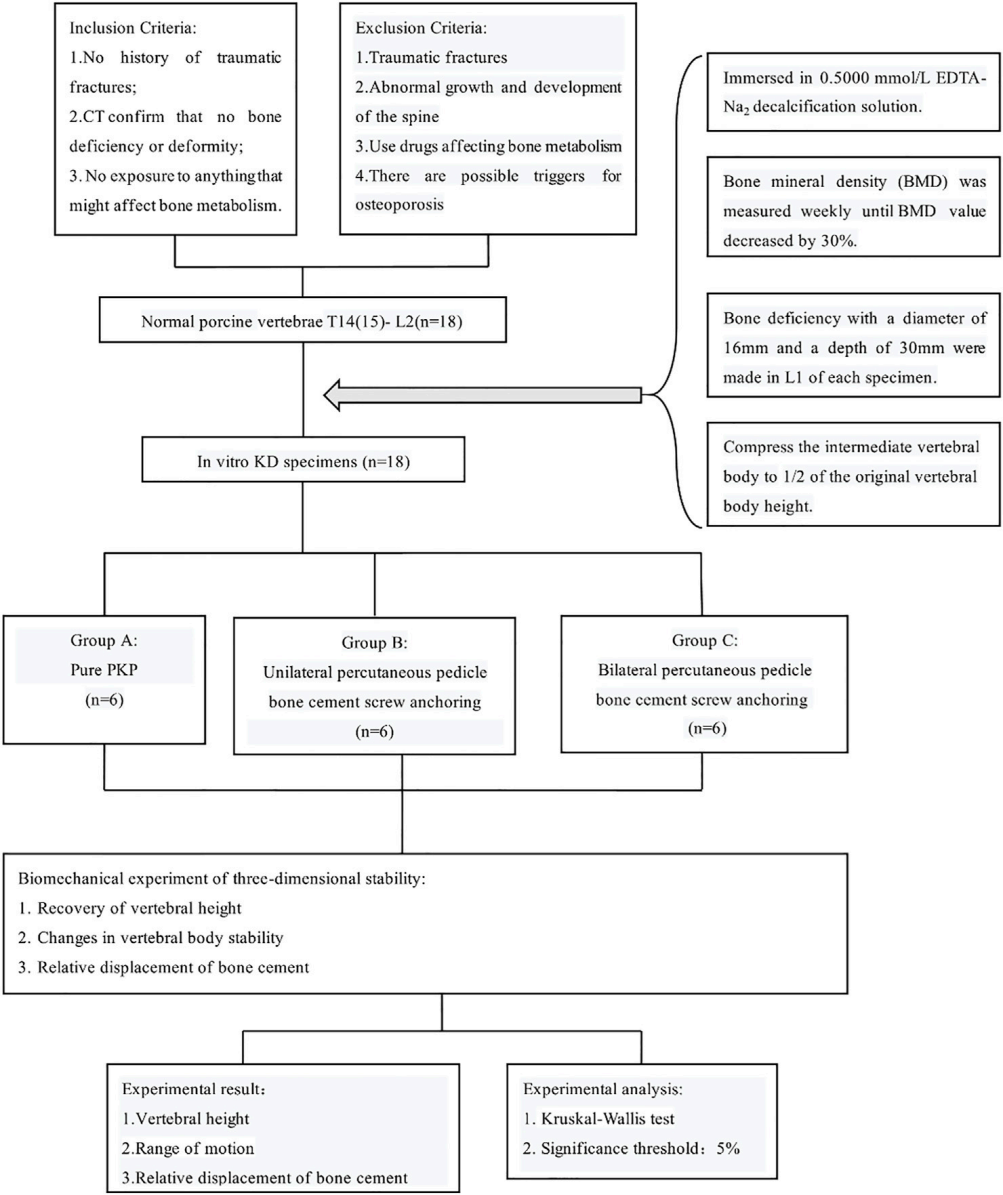
Flow chart of the study design.

This study was performed using 18 thoracolumbar vertebrae T14 (T15) -L2 harvested from mature pigs (weight 110–130 kg). The porcine thoracic vertebrae have 14–15 segments and the lumbar vertebrae have 6–7 segments. Prior to their use in the study, the pigs were healthy and not exposed to any environmental factors that could affect their bone quality. All specimens were rigorously inspected to ensure that there were no defects. CT scan in the imaging department of Tianjin Hospital confirmed that the structure of them was intact and no deficiency (Figure 2). The paravertebral muscles and other soft tissues on both sides of the specimen were removed, and the intervertebral disc, spinous process, interspinous ligament, posterior longitudinal ligament, facet joint, ligamentum flavum and transverse process were preserved. Rinse with water and soak in 10% formaldehyde solution for 24 h. The study design and subjects are presented in Figure 3.

### 2.2 Preparation of KD specimens

**FIGURE 4:**
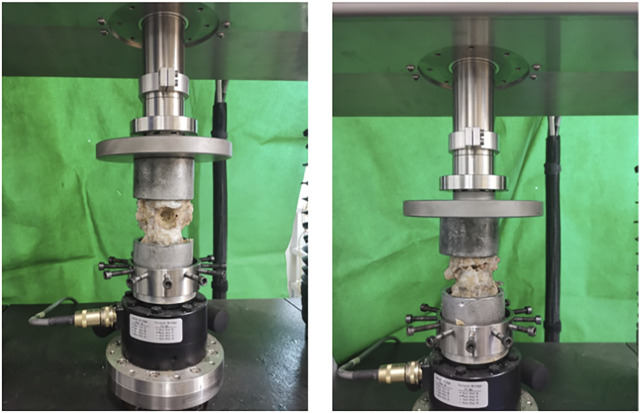
Compression process of specimens by biomechanical testing machine (ElectroForce 3510, Bose).

The 18 triple vertebrae specimens were completely immersed in 0.5000 mmol/L EDTA-Na_2_ decalcification solution (pH 7.3 ± 0.1) (Lee et al., 2011; Hsieh et al., 2020; Liu JB. et al., 2022). Bone mineral density (BMD) of all vertebral bodies was measured weekly and the solution was changed until the percentage loss of BMD value reached 30%. After all specimens met the criteria of osteoporosis, a conical vertebral bone defect with a diameter of 16 mm and a depth of 30 mm was created in L1 of each specimen using a grinding drill. The animal model of KD was prepared by smearing the cancellous bone surface in the cone-shaped defect with bone wax to simulate the hardened bone at the defect site. L1 of each specimen was compressed to 1/2 the height of the original vertebral body by biomechanical testing machine (ElectroForce 3510, Bose) (Figure 4).

### 2.3 Surgical methods and experimental grouping

18 triple vertebrae specimens were randomly divided into 3 groups, which were recorded as group A, B, and C (*n* = 6/group). Group A underwent pure PKP; Group B underwent unilateral pedicle cement screw fixation combined with PKP; Group C underwent bilateral pedicle cement screw fixation combined with PKP.

The details of PKP surgical were performed based on standard procedure (Lei et al., 2020). The puncture position was adjusted according to the IVC to ensure that the tip cone was located in the cleft. The tip cone was withdrawn, and the guide drill was inserted in sequence to a distance of approximately 3–5 mm from the posterior edge of the vertebral body. The collapsed vertebral tissue was opened with a balloon to create a cemented cavity. Apply the PKP working sleeve to push the prepared bone cement along the vertebral arch in slow and staged injections, stopping the procedure immediately if high resistance is encountered or if the bone cement is close to the posterior wall of the vertebral body. The amount of bone cement was selected as 8 mL according to the size of the porcine vertebrae, and the distribution of the bone cement in the vertebral bone was observed. The curing time of the bone cement was 15 min. After waiting for sufficient time and confirming the curing of the bone cement, the pusher and working cannula were rotated and then withdrawn.

The following were details of the surgical procedure for novel pedicle bone cement screw fixation combined with PKP. The surface projections of the bilateral pedicle margin of the diseased vertebra were used as the pedicle screw entry point. Firstly, 1.5 mL bone cement was pushed in with the bone cement pusher, and appropriately sized pedicle bone cement screw were placed without penetration from the anterior cortical aspect. Bone cement was injected along this pedicle screw so that so that the cement flowing out of the anterior and lateral holes of the pedicle screw fused with the previously injected cement and became a single unit. After the cement had set, and the tail of the pedicle screw was broken.

**FIGURE 5:**
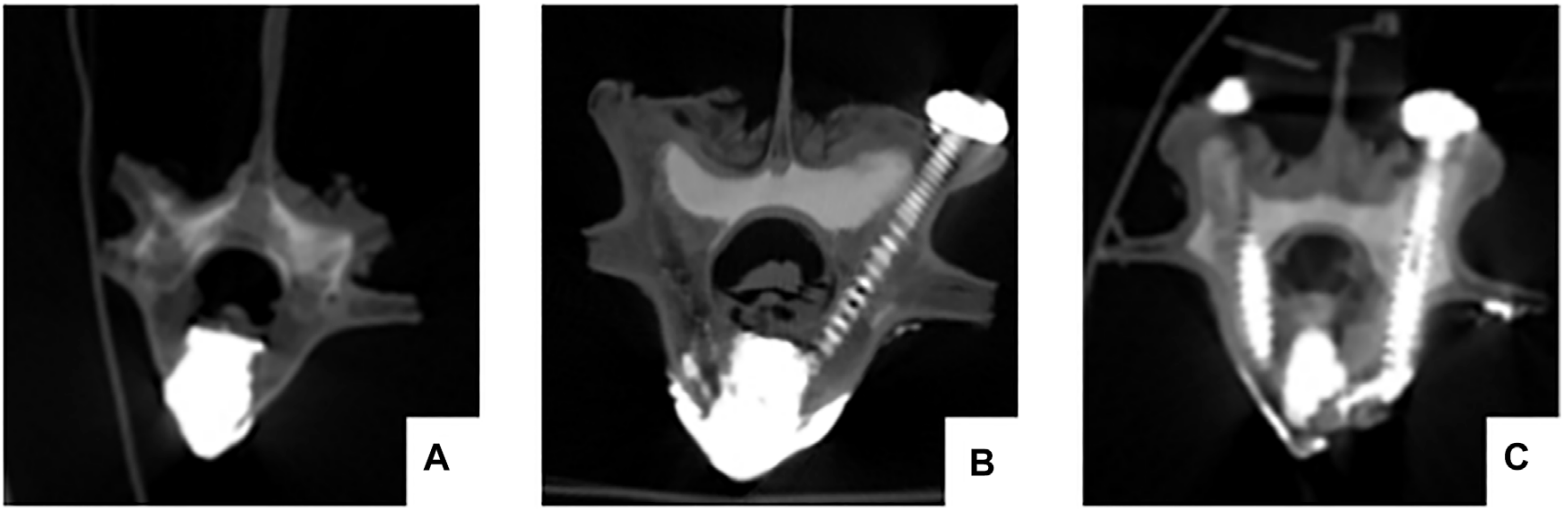
The postoperative CT scan shows satisfactory bone cement filling and accurate placement of the pedicle screws. No vertebral damage or spinal cord compression occurred during the operation. **(A)** pure PKP; **(B)** unilateral pedicle cement screw fixation combined with PKP group; **(C)** bilateral pedicle cement screw fixation combined with PKP group.

CT was performed on all specimens immediately after surgery. Observation of cement filling, screw placement, vertebral body injury and spinal cord compression (Figure 5).

### 2.4 Three-dimensional stability biomechanical experiments

**FIGURE 6:**
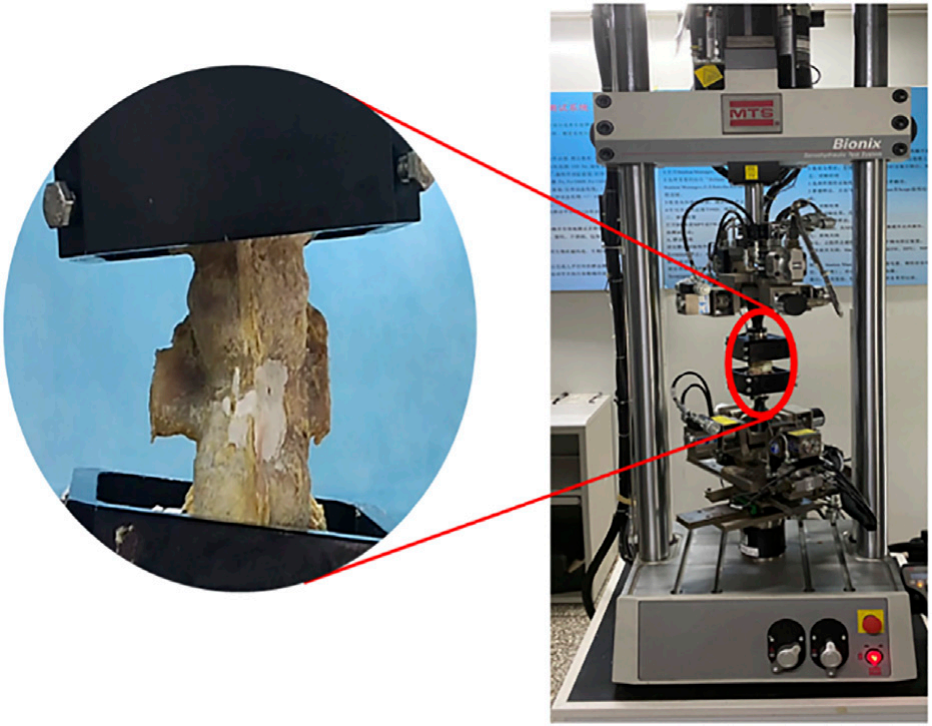
Three-dimensional stability biomechanical experiments.

The upper and lower vertebrae of each specimen were embedded with denture base resin without crossing the intervertebral disc at depth. And in the procedure, try to make the part fixed to the MTS multi-degree of freedom simulation test system (MTS, Bionix 370.02, Figure 6) as regular in shape as possible, so that the specimens were sufficiently fixed during movement and did not become skewed. The upper and lower bases of each specimen were tightly attached to respective bases of the test machine with the T12/L1 and L1/L2 discs in a horizontal neutral position. The specimens were preloaded at a rate of 1 mm/min and stopped when the force reaches 100 N. The purpose was to eliminate creep movement and to make the vertebrae fully fixed in the mold so that they would not slip off and affect the determination of the value. The test force was set to zero after adequate fixation was completed. An axial load of 500 N was applied to simulate the mass of the upper body. The specimens were kept moist by spraying physiological saline every 5 min during the test to reduce the error caused by evaporative water loss.

#### 2.4.1 ROM testing of vertebrae

By adjusting the tightness of the screws fixing the specimen until the constraint force in each direction was shown as 0 on the sensor. The 5 Nm force was then applied to the specimen to complete the flexion, extension, left/right lateral flexion and left/right axial rotation, and the maximum activity angle was recorded.

#### 2.4.2 Bone cement relative displacement test

Each postoperative specimen underwent movements with the same force applied at maximum angles of 5° and 10° in each direction, completing each movement six times. Set the angular speed to 0.003 rad/s. The specimens were scanned by CT after movement to observe the relative displacement of the bone cement.

### 2.5 Statistical analysis

SPSS 26.0 software (IBM, United States) was used for statistical analysis. The means and SD ( $\bar{X}$ ±s) were calculated for BMD during specimen decalcification. Medians and interquartile range [M(IQR)] were calculated for the data on the height of the vertebral body, ROM and bone cement displacement in each of these groups. The Kruskal–Wallis test was used for the comparative analysis of experimental results among different groups. The 5% significance threshold indicated a difference.

## 3 Results

### 3.1 BMD during specimen decalcification

**FIGURE 7:**
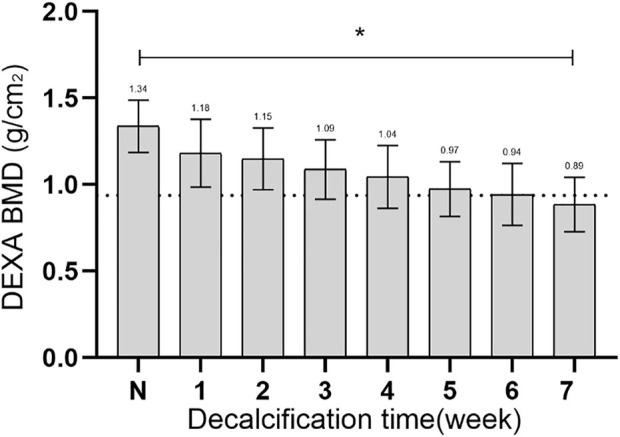
The changes in BMD from normal to osteoporotic status. N, the initial BMD; *, statistical differences.

The changes in regional bone mineral density (BMD) from normal to osteoporotic status were shown in Figure 7. The initial BMD was recorded as 1.34 ± 0.15 g/cm^2^, and after being treated with EDTA for 7 weeks, the BMD decreased to 0.89 ± 0.16 g/cm^2^. After EDTA treatment for 7 weeks, the BMD decreased to below 30% of the normal vertebral value (*p* < 0.05), indicating the onset of osteoporosis in the experiment.

### 3.2 The recovery status of vertebral height

The vertebral heights at 500 N of axial compression in each group were shown in Table 1. There was no statistical difference in vertebral body height before treatment (*p* > 0.05) and after treatment (*p* > 0.05). After different treatments, all three groups had higher vertebral heights than before surgery (*p* < 0.05).

**TABLE 1:** M(IQR) of the vertebral heights (mm) at 500 N of axial compression in each group, *n* = 6.

Group	Pre-treatment	Post-treatment	Wilcoxon signed-rank test	
			Z-value	*p*-value
A	121.02 (23.66)	131.06 (16.99)	1.782	0.075
B	109.27 (32.04)	121.84 (31.91)	2.201	0.028
C	121.85 (25.96)	137.36 (24.17)	2.201	0.028
H-value	0.881	1.205		
*p*-value	0.644	0.548		

### 3.3 Range of motion

**FIGURE 8:**
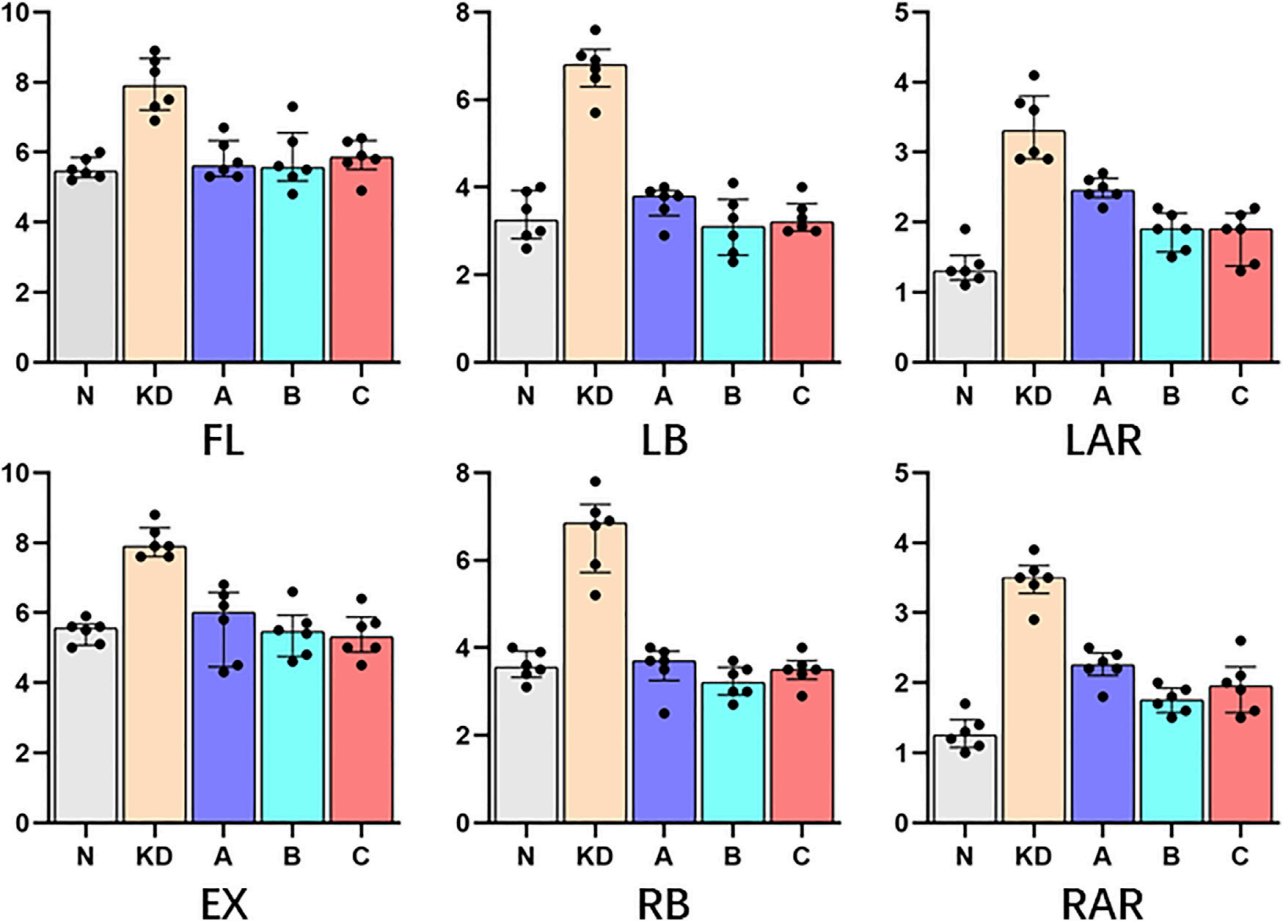
ROM (°) were indicated as the medians and interquartile range [M(IQR)].

ROM measured at 5 Nm in six directions for intact vertebral specimens (N), KD specimens (KD), postoperative group A (A), postoperative group B (B), and postoperative group C (C) were shown in Figure 8; Table 2. Statistical analysis showed that the ROM of the KD specimens was greater (*p* < 0.05) compared to the N control group, except for right lateral bending. In the left/right axial rotation, the specimens were still significantly different (*p* < 0.05)from the intact specimens in terms of ROM after pure PKP.

**TABLE 2:** ROM (°) was shown with M (IQR); “N”, intact lumbar spine; “KD”, KD specimens; “A”, PKP; “B”, unilateral pedicle cement screw fixation combined with PKP; “C”, bilateral pedicle cement screw fixation combined with PKP. FL, flexion; EX, extension; LB, left lateral bending; RB, right lateral bending; LAR, left axial rotation; RAR, right axial rotation. *, statistical difference with “N”; ▲, statistical difference with KD.

	N (*n* = 6)	KD (*n* = 6)	A (*n* = 6)	B (*n* = 6)	C (*n* = 6)
FL	5.45 (0.57)	7.90 (1.48)*	5.60 (1.03)▲	5.55 (1.38)▲	5.85 (0.83)
EX	5.55 (0.60)	7.90 (0.83)*	6.00 (2.13)	5.45 (1.17)▲	5.30 (1.00)▲
LB	3.25 (1.10)	6.80 (0.85)*	3.80 (0.57)	3.10 (1.27)▲	3.20 (0.63)▲
RB	3.55 (0.60)	6.85 (1.55)	3.70 (0.67)	3.20 (0.63)▲	3.50 (0.43)▲
LAR	1.30 (0.35)	3.30 (0.90)*	2.45 (0.27)*	1.90 (0.55)▲	1.90 (0.75)▲
RAR	1.25 (0.40)	3.50 (0.40)*	2.25 (0.32)*	1.75 (0.35)▲	1.95 (0.65)

### 3.4 Relative displacement of bone cement

**FIGURE 9:**
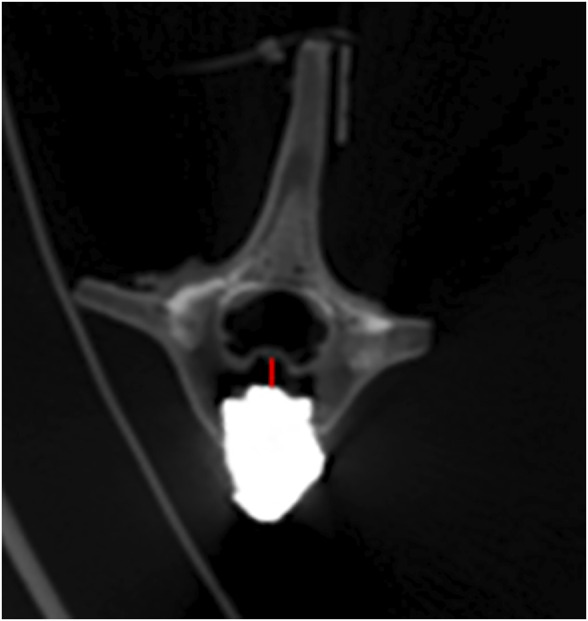
Bone cement displacement after movement. The red line represents the maximum displacement distance.

All specimens underwent 6 times of activity with different deflection angles in 6 directions of flexion, extension, left/right lateral bending, left/right axial rotation. After repeated movement with 5° as the maximum activity angle, no significant displacement of bone cement was seen in all vertebrae on the CT images. After repeated movement with 10° as the maximum activity angle, the relative displacement of the bone cement in each group is shown in Table 3, and the partial post-activation CT of the bone cement is shown in Figure 9. Compared with pure PKP, pedicle cement screw fixation combined with PKP can significantly reduce the relative displacement of the bone cement (*p* < 0.05).

**TABLE 3:** Relative displacement (mm) of bone cement with 10° as the maximum activity angle. “*”, statistical difference (*p* < 0.05) compared to the A group.

Group	M (P_25_, P_75_)	Kruskal–Wallis test	
		H-value	*p*-value
A	3.50 (1.50,6.48)	8.307	0.016
B	0.00 (0.00,0.55)*		
C	0.00 (0.00,0.43)*		

## 4 Discussion

With the aging of society, osteoporosis has become the most common bone metabolic disease which is characterized by low bone density and leaving the affected bones susceptible to fracture (Song et al., 2022). KD is a complication or end-stage manifestation of vertebral compression fractures and once it occurs, it can seriously affect the patient’s quality of life and survival, requiring more aggressive surgical treatment (Zhang et al., 2022; Gou et al., 2023). With the leap forward in minimally invasive spine technology over the last decade, the traditional open fixation-fusion surgical approach is not the best choice for patients with no nerve compression symptoms (Huang et al., 2018). PVP and PKP are more widely used in clinical practice because of its advantages in terms of economic cost, operative time, blood loss, and radiation exposure. However, they have some postoperative risks, such as loosening of the cement mass, displacement, and fracture of the cement mass, resulting in adverse consequences such as neurological impairment and re-aggravation of the posterior convexity deformity.

**FIGURE 10:**
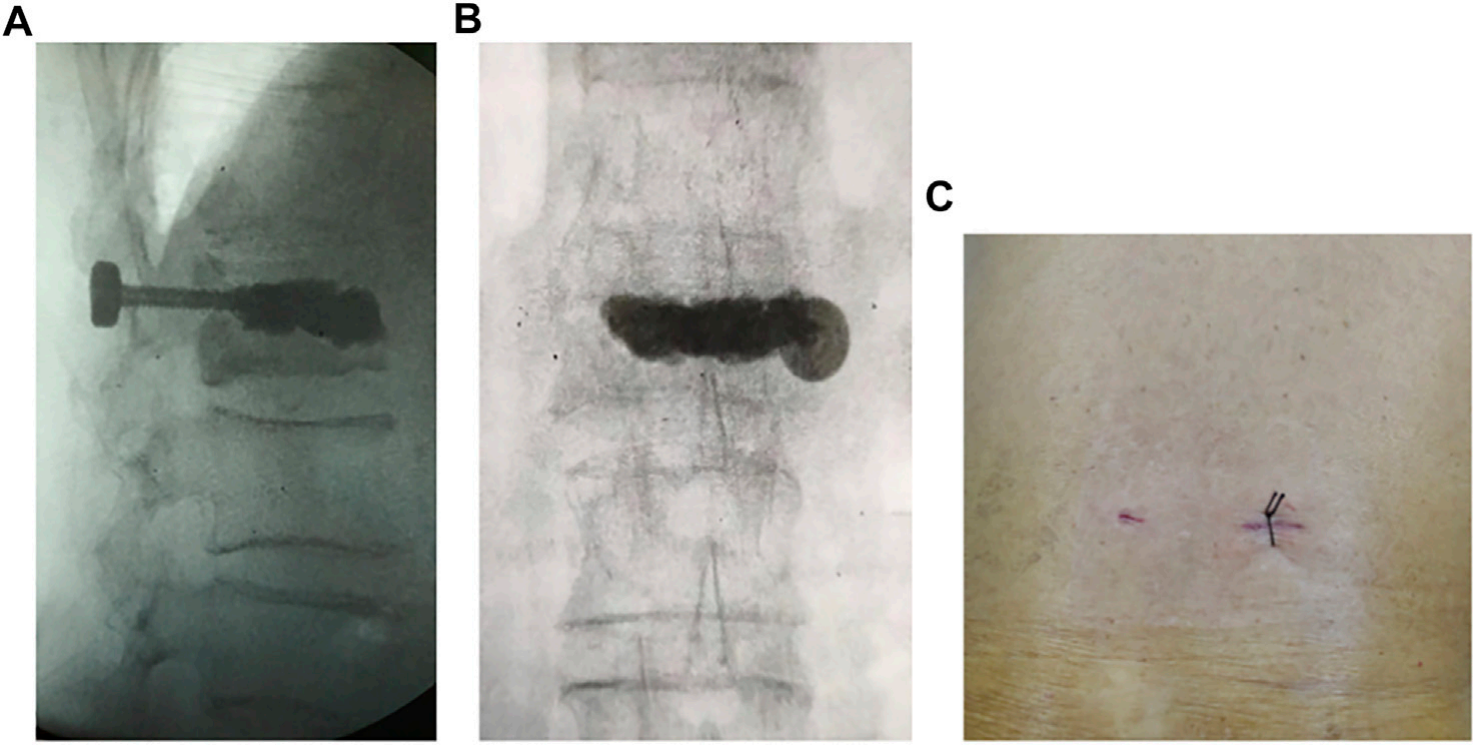
Intraoperative fluoroscopic and incisional photograph of a patient with KD treated with a novel minimally invasive pedicle bone cement screw: **(A)** lateral view **(B)** anteroposterior view **(C)** incisional view (1.5 cm).

There is a significant technical gap in the current approach to reduce postoperative cement displacement in KD. In order to solve these clinical problems, pedicle bone cement screw can act as a “bridge” to link the bone cement with the surrounding bone tissue while being placed percutaneously and minimally invasively, reducing the incidence of bone cement loosening and displacement. It can be implanted into the diseased vertebra through the puncture needle used in the PKP/PVP treatment of KD, without causing additional trauma (Figure 10). The front end of the pedicle screw is equipped with multiple bone cement overflow hole, which can directly inject bone cement and fuse with the bone cement injected by PKP/PVP, ensuring a strong and anchored connection between the screw and bone cement. Meanwhile, the screw provides a fixed point of stability for the bone cement in the vertebral body. By connecting to the pedicle, the screw can anchor the bone cement in place, preventing it from loosening or displacing over time.

### 4.1 Preparation of KD specimens

Biomechanical testing of new spinal implants *in vitro* is essential for their safety and efficacy (Lee et al., 2011). However, the limited availability of human cadavers leads to inconsistent specimen quality, making it difficult to obtain enough specimens for controlled experiments to explore the effects of these implants (Lee et al., 2011; Hsieh et al., 2023). The vertebrae of other large mammals are morphologically and biomechanically different from the human vertebrae, and their experimental parameters are not directly transferable to the human spine. However, due to the similarity of the porcine spine to the human in terms of size, nutritional make-up, bone structure, and mineral metabolism, it has been frequently used in some studies to verify the effectiveness of spinal fusion and internal fixation, and to a certain extent to draw clinically relevant conclusions (Lee et al., 2011; Hsieh et al., 2020; Laznovsky et al., 2022).

To model osteoporosis, demineralization can be performed using reagents such as hydrochloric acid and hydrogen peroxide, but the models prepared using these methods differ from the pathological process of human osteoporosis, which is more like osteomalacia (Elfar et al., 2014; Stewart et al., 2020). EDTA is a chelating agent that binds to the metal ion Ca^2+^ and acts more slowly, preserving the natural biological structure of collagen better than the stronger and faster acting hydrochloric or nitric acids (Lee et al., 2011). After 7 weeks, the difference in BMD values of the vertebrae before and after decalcification was more than 30%. Since there is currently no investigation data on peak BMD in porcine spine, the percentage of BMD loss was measured using the human osteoporosis standard, where 25% loss is considered an indicator of osteoporosis (Lee et al., 2011; Siris et al., 2014).

In the preparation of *in vitro* osteoporotic specimens, treatment with 10% formaldehyde solution for 24 h was a common approach in previous biomechanical experimental studies because of the prolonged exposure to ambient temperature (Lee et al., 2011; Hsieh et al., 2022). This preservative measure inevitably causes changes in the biomechanical properties, focusing particularly on Young’s modulus and impact energy in short time applications (Currey et al., 1995; Holewijn et al., 2017). This alteration makes it necessary to use smaller moments and slower angular velocities in the experiments to avoid damaging the specimens, which can also affect the experimental results to some extent.

### 4.2 Recovery of vertebral body height

In clinical practice, it has been found that KD occurs most commonly at the thoracolumbar junction, which is also the common location for osteoporotic vertebral fractures. From a biomechanical perspective, one important reason for the development of KD from vertebral microfractures is that the shear stress in the thoracolumbar segment changes after a large range of motion (Li et al., 2011). Following vertebral fracture, the vertebral morphology changes, mainly characterized by loss of vertebral height and spinal kyphosis (Röllinghoff et al., 2013). In this study, we found that satisfactory vertebral height was achieved regardless of the use of pedicle bone cement screw or not. This result is similar to the clinical treatment outcomes reported by Noriega et al. (2019) (Wang et al., 2021). It can be concluded that using the novel type of pedicle screw combined with PKP for the treatment of KD can achieve good effectiveness in static standing position. The restoration of vertebral height can prevent spinal center of gravity shifting, which in turn restores the internal biomechanical environment of the vertebrae and reduces the risk of nonunion (Cao et al., 2020; Najjar et al., 2023).

Both PVP and PKP are commonly used for vertebral compression fractures. Patients with severe OVCF can be treated with PVP in a prone position with the chest and hips resting on soft pillows to maintain spinal hyperextension to help restore vertebral body height (Chin et al., 2006; Shin et al., 2009; Yu et al., 2020). Since this experiment used an *in vitro* specimen, we had to use a balloon to expand the vertebral body when repairing the vertebral height.

### 4.3 Changes in vertebral body stability

After making the specimens into IVC-like vertebrae, ROM was significantly increased compared to intact vertebrae under 5 Nm torque. In the experiment, we observed that the increased instability mainly occurred at the site of the discontinuous cortical bone. Restoring the stability of the vertebral body is also a clear need in the treatment of KD (Yokoyama et al., 2013). The ROM of the vertebral body is an important parameter of vertebral stability. According to Denis’ three-column theory, recompression of the spine may be influenced by spinal stability (Denis, 1983). Low spinal stability may lead to an increased risk of recompression. In this study, filling with bone cement reduced the maximum displacement of the KD specimens and approached the intact vertebrae, which was also demonstrated by some related studies (Dai et al., 2022). However, in the PKP group, the specimens still differed from the intact vertebrae in terms of stability during rotation. This result suggests that the stability of the vertebral body can be further increased by pedicle screw fixation, thus reducing the incidence of recompression.

### 4.4 Relative displacement of bone cement

An important purpose of PKP and PVP is to return the patient to normal living conditions as soon as possible, which requires that the patient be able to perform the angle required after surgery. It is also required to reduce the probability of bone cement displacement during this period (Qi HR. et al., 2022). The gold standard in biomechanical testing is to apply moments to the specimens (Wilke et al., 1998). However, due to the *in vitro* osteoporotic specimens we used, which was more flexible than undecalcified specimens, this torque may result in a significant movement of the lumbar spine that should not have been accomplished. In previous studies, maximum rotational value was similar, with a range of 4.05° to 7.10° for lying and 9° to 14° for standing (Yeager et al., 2014; Breen et al., 2019; Daniel et al., 2023). In consideration of the maximum ROM of L12-L2 and avoiding excessive damage to the experimental specimen during the experiment, 5° was used to simulate ROM for lying and 10° to simulate ROM of standing (Yeager et al., 2014; Breen et al., 2019; Daniel et al., 2023).

Previous clinical case reports have also shown displacement of bone cement, but our experimental results generally have a higher displacement rate (Korovessis et al., 2013), perhaps due to the extreme smoothness of the cortical bone defect and the complete coverage of the cancellous bone surface by bone wax in our experimental models to better control the number of variables. The KD model designed in the experiment meets the clinical and radiographic characteristics of intravertebral cleft (Formica et al., 2016; Qi J. et al., 2022). This makes it possible that the measured displacement of the bone cement in the experiment indicates the trend and magnitude of displacement. The screw can release bone cement through the lateral hole at the front end of the screw, thoroughly filling the cracks in the vertebral body, so that the bone cement and the screw are connected as a whole. At the same time, the screw connects the strongest part of the bone at the pedicle, and ultimately reinforces the interaction between the bone cement and the surrounding bone tissue as a “bridge”. This achieves the function of preventing the bone cement from loosening and displacing.

### 4.5 Study limitations

The experiment has convincingly demonstrated that the novel bone cement screws provide excellent fixation of bone cement masses, providing them with less mobility and more stability. However, the experiment has certain limitations. First, the specimens used were porcine spines prepared by formaldehyde solution and EDTA, which inevitably led to altered the soft tissue properties and thus the overall biomechanical properties and therefore cannot be identical to human specimens. Second, the number of specimens was small, and more data are needed to support the results. Third, during the experiments, shear forces were still unavoidable, with a maximum value of 0.3 Nm. To obtain more reliable test results, these forces had to be reduced further, even to pure moments. Finally, the bone cement relative displacement was linked to the ROM, which is not correspond to the common recommendations in previous studies.

## 5 Conclusion

In an *in vitro* animal vertebral body model, the application of a new minimally invasive cemented screw for Kümmel’s disease meets the need for restoration of vertebral body height. It also possesses a stronger effect of fixing the vertebral body and limiting bone cement displacement compared to pure PKP. From the viewpoint of biomechanics, both unilateral and bilateral anchoring have better effects, which provides strong evidence and guidance for later clinical application.

## Data Availability

The raw data supporting the conclusion of this article will be made available by the authors, without undue reservation.
